# Spatial and temporal variation of genetic diversity and genetic differentiation in *Daphnia galeata* populations in four large reservoirs in southern China

**DOI:** 10.3389/fmicb.2022.1041011

**Published:** 2022-11-11

**Authors:** Qi Huang, Lei Xu, Lili Xie, Ping Liu, Eric Zeus C. Rizo, Bo-Ping Han

**Affiliations:** ^1^Department of Ecology and Institute of Hydrobiology, Jinan University, Guangzhou, Guangdong, China; ^2^South China Sea Fisheries Research Institute, Chinese Academy of Fishery Sciences, Guangzhou, Guangdong, China; ^3^College of Environmental Science and Engineering, Yangzhou University, Yangzhou, Jiangsu, China; ^4^Division of Biological Sciences, College of Arts and Sciences, University of the Philippines-Visayas, Miagao, Iloilo, Philippines; ^5^Engineering Research Center of Tropical and Subtropical Aquatic Ecological Engineering, Ministry of Education, Guangzhou, China

**Keywords:** *Daphnia*, genetic differentiation, genetic erosion, fine-scale, environmental selection, seasonal dynamics

## Abstract

*Daphnia galeata* is a common and dominant species in warmer waters, and has a strong top-down effect on both phytoplankton and bacteria. The knowledge of its temporal and spatial patterns of genetic diversity is fundamental in understanding its population dynamics and potential ecological function in ecosystems. Its population genetics have been investigated at regional scales but few within regions or at smaller spatial scales. Here, we examined the fine-scale spatial genetic variation of *D. galeata* within four large, deep reservoirs in wet and dry seasons and the six-year variation of genetic diversity in one of the reservoirs by using cytochrome c oxidase subunit I and microsatellites (simple sequence repeat). Our study shows that fine-scale spatial genetic variation commonly occurred within the reservoirs, indicating strong environmental selection at least in the two of reservoirs with strong longitudinal gradients. Since the environmental gradients established in the dry season was largely reduced in the wet season, the fine-scale spatial genetic variation was much higher in the dry season. The dynamics of local genetic diversity did not follow the theoretical pattern of rapid erosion but peaked in mid or mid-late growth season. The local genetic diversity of *D. galeata* appears to be shaped and maintained not only by recruitment from resting egg banks but also by gene flow within reservoirs. The temporal and fine-scale genetic variation within a water body suggests that it is necessary to pay attention to sampling periods and locations of a given water body in regional studies.

## Introduction

Spatial patterns of biological composition and diversity are a major topic in modern ecology, providing core knowledge for conserving biodiversity and ecosystem management (Mittelbach, 2012). Species composition of a local community is determined not only by local processes such as biological interaction, adaptation, and stochastic variation but also by regional processes of species dispersal and geographic isolation and even species speciation (Ricklefs, 1987, 2004). There are two frameworks for viewing the composition of biological communities (Tilman, 2004). In niche theory, species composition is mainly determined by environmental or ecological selection (Tilman, 1994). In neutral theory, in contrast, species composition is determined by random drift and dispersal limitation or mass effects (Hubbell, 2001, 2006). In the two frameworks, species are differently assumed to be under selection or neutral. If species are equal or neutral, spatial patterns can be only observed in high dispersal species groups on a large scale. Due to their size, most plankton groups can be passively and highly dispersed through airborne and waterborne ways (Leibold and Norberg, 2004; Louette and De Meester, 2005; Incagnone et al., 2015). However, their spatial patterns have been observed both across water bodies and within a water body (Grosbois et al., 2016; Rizo et al., 2020). Such patterns strongly suggest that environmental filtering and selection play a role in shaping species composition and diversity.

Spatial patterns of genetic variation or diversity are structured in ways similar to those for patterns of species diversity at the community level (Gillespie, 2004; Hamilton, 2009; Haileselasie et al., 2018). Intraspecific genetic structure commonly occurs between populations with limited gene flow or under strong selection. Fine-scale population structure has been observed for amphibians and fishes in a single water body or a single river basin (Wagner and McCune, 2009; Triest et al., 2014). Biological traits under selection strongly influence spatial genetic structure (Vekemans and Hardy, 2004). However, not all species have conspicuous morphological and behavioral traits that can easily be observed and/or measured. For example, many planktonic organisms have distinct traits at the genus level but quite similar traits at the species level within the genus (De Meester, 1997; Gómez et al., 2000, 2002). Although high-resolution markers such as microsatellites (or SSR: Simple Sequence Repeat) and SNPs have been developed to reveal population subdivision at the regional scale, there are few studies on the intraspecific genetic structure of plankton at small scales (Weider, 1985; Petrusek et al., 2013). Nevertheless, plankton species have specific traits (e.g., physiological traits) that are hard to be observed. Despite high dispersal, fine-scale population structure has been observed for zooplankton in some harsh and highly heterogeneous environments (Carvalho and Crisp, 1987; Petrusek et al., 2013). Spatial heterogeneity of plankton communities is common within many large waterbodies that have strong environmental heterogeneity (Grosbois et al., 2016; Rizo et al., 2020). If used genetic markers are not totally neutral, i.e., slightly under selection, population genetic differentiation could be observed at a local scale, even between adjacent sampling sites within a water body (Petrusek et al., 2013; Frisch et al., 2021).

*Daphnia* is one of the largest genera in Cladocera with more than 100 species found around the world (Forró et al., 2008). They represent the most important herbivores in natural and man-made lakes and ponds (Seda and Petrusek, 2011). Most species of *Daphnia* reproduce by cyclic parthenogenesis and produce resting eggs that accumulate in the sediments as a seed bank (De Meester et al., 2006). As accumulated in time and space, a resting egg bank contains diverse genotypes and hatching asynchronism of different genotypes influences observed genetic diversity (Brendonck and De Meester, 2003; Hulsmann et al., 2012). Like spatial patterns observed for zooplankton at the community level, a similar pattern for genetic composition (or diversity) is possible for *Daphnia* species at the population level when high-resolution markers [such as SSR and cytochrome c oxidase subunit I (COI), etc] are used (Yin et al., 2012; Petrusek et al., 2013; Liu et al., 2022).

*Daphnia galeata* is a pelagic species common in both Europe and Asia and occurs in eutrophic and warmer water (Stich and Lampert, 1984). It showed significant genetic differentiation along both vertical and longitudinal gradients in Rimov reservoir, Czech Republic (Seda et al., 2007; Macháček and Seda, 2008; Yin et al., 2012; Petrusek et al., 2013). *D. geleata* is also common in waters of tropical and subtropical China (Han et al., 2012; Liu et al., 2019; Ma et al., 2019), where it is subject to high and year-round predation pressure. To be persistent and dominant in warmer waters, *D. geleata* needs some ecological mechanisms to maintain its genetic diversity. In tropical and subtropical regions, the cold period usually have a water temperature of 8–15°C around “late winter” and the beginning of spring required for the hatching of *D. geleata’*s resting eggs is relatively short. Therefore, both spatial patterns and seasonal dynamics of genetic diversity within tropical and subtropical water bodies may be different from temperate regions. In tropical and subtropical reservoirs of southern China, strong and stable environmental gradients that are commonly established in dry seasons can be largely reduced in wet seasons due to high water flow during monsoonal periods. In this study, we hypothesize that *D. galeata* in warmer waters: 1) has a clear spatial pattern of genetic diversity within a heterogeneous water body, and 2) has a genetic diversity that peaks in early spring when the species hatch from the sediments and then is quickly eroded. To test the assumptions, we collected *D. galeata* along a longitudinal gradient from four large reservoirs in tropical and subtropical China for two seasons, and the populations in their growing season in a tropical reservoir for six successive years. *Daphnia galeata* individuals were sequenced for COI and SSR to measure the genetic diversity of populations.

## Materials and methods

### Sample collection

**Figure 1:**
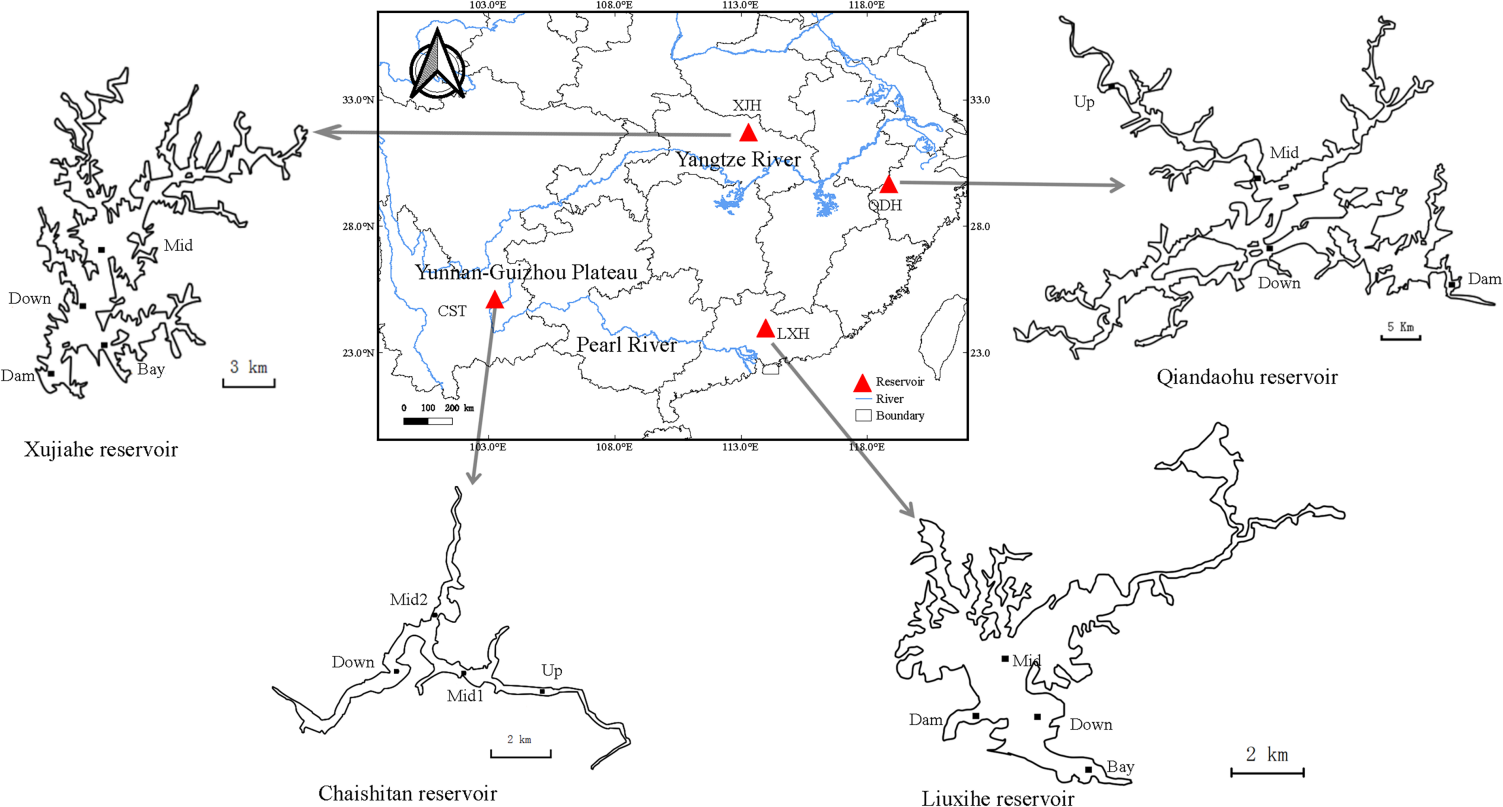
Sampling sites in each of four investigated reservoirs. Each reservoir has four sampling sites, Up: upstream; Mid: midstream; Down: downstream, Dam: near the dam. Two reservoirs in Yangtze River Basin: Qiandaohu reservoir (QDH) and Xujiahe reservoir (XJH); Two reservoirs in Pearl River Basin: Chaishitan reservoir (CST) and Liuxihe reservoir (LXH). Only Chaishitan and Qiandaohu reservoirs were sampled in the riverine zone (Up), where it was deep enough for *Daphnia galeata* to establish a stable population.

*Daphnia galeata* were collected from four large and deep reservoirs in southern China (Figure 1): Qiandaohu reservoir (29.61°N, 118.96°E) and Xujiahe reservoir (31.57°N, 113.62°E) in the Yangtze River Basin, Chaishitan reservoir (24.98°N, 103.32°E) and Liuxihe reservoir (23.75°N, 113.79°E) in the Pearl River Basin. The Chaishitan reservoir is located on the Yunnan-Guizhou Plateau and has a climate similar to that in the Yangtze River Basin. Chaishitan and Qiandaohu reservoirs have a typical longitudinal environmental gradient, i.e., riverine, transitional and lacustrine zone. Each reservoir was sampled from four sites in the wet (summer) and dry (spring or autumn) seasons of 2016 (Supplementary Table 1), which covers heterogeneous habitats. We obtained 32 seasonal populations from the four reservoirs. In subtropical reservoirs, the population of *D. galeata* had a growing season from early spring to late summer. To observe the dynamics of genetic diversity and clone erosion, *D. galeata* was sampled at the central pelagic zone of Liuxihe reservoir from 2012 to 2017. The sampling was conducted every 15 days during the growing season in 2012 and 2013, and every 30 days during the growing season in 2014–2017. Only two samples were collected in May and June of 2016, and excluded from the analysis. Finally, we also obtained 32 temporal populations in Liuxihe reservoir. Individuals of *D. galeata* were harvested using a 110 μm vertical plankton net and the samples were fixed with 75% ethanol in the field. All *Daphnia* individuals were picked out under a dissecting microscope (Olympus: SZXZ-ILLB) and identified under an optical microscope (Olympus U-LH100-3) according to criteria given by Benzie (2005) and further confirmed by barcoding. Samples were preserved in 95% ethanol and stored at-20°C for DNA extraction and sequencing.

### DNA extraction and PCR amplification

The genomic DNA was extracted using an Ultra-Sep Gel Extraction Kit (Omega, USA). Individuals of *D. galeata* were picked out from 95% ethanol, rinsed repeatedly with double-distilled water, and transferred individually to a 500 μl tube. We added 200 μl Lysis buffer and 4 μl Protease K (20 mg·ml^−1^). The mixture was vortexed and subsequently incubated for 2 h at 55°C. Next, 100 μl of chloroform-isoamyl alcohol (24:1) was added and the mixture was centrifuged vigorously at 10,000 rpm at 10°C for 10 min. The supernatant was moved to a new 500 μl tube and added 3 μl beads. After adsorption for 2 min, we added 300 μl Binding buffer and centrifuged at 10,000 rpm for 4 min at 10°C. The supernatant was discarded and the precipitate was washed with 300 μl washing buffer and centrifuged at 10,000 rpm and 10°C for 4 min. Then the supernatant was removed and the precipitate was left to dry at room temperature for 2–3 h. After that, 25 μl Elution buffer was added to the supernatant, which was placed 4°C for 12 h, and centrifuged at 10,000 rpm and 10°C for 4 min. Finally, the supernatant was obtained and stored at −20°C.

The individual DNA materials were first used for the microsatellite data and the remaining materials were used for sequencing COI. For each population, 32 individuals were randomly picked. DNA amplification was performed by polymerase chain reaction using nine microsatellite markers developed for species of *Daphnia* in Europe (Brede et al., 2006). The nine microsatellite markers were used, including SwiD5, SwiD7, SwiD10, DaB17, DaB10, SwiD2, SwiD12, SwiD18, and Dgm113. The microsatellite primers were synthesized by Invitrogen, and forward primers were labeled with the FAM fluorophore. The total reaction volume (30 μl) contained 10 × buffer (Mg^2+^plus), 0.25 mM dNTP Mixture 2.5 mmol/l each, 20 μM each primer, 0.5 U Taq (TaKaRa), and 3 μl template DNA. The PCR reaction procedure is used as follows: pre-denaturation at 95°C for 5 min, 35 cycles of 95°C for 30 s, annealing at 55°C for 45 s, extension at 72°C for 30 s, the final extension at 72°C for 3 min, and the reaction ended at 4°C. Polymorphism was assessed on an ABI PRISM 3730 capillary DNA sequencer, using an internal Liz Gene-scan size standard (Applied Biosystems). Amplified fragments for all primers contained between 99 and 234 nucleotides. Genotyping was checked by GeneMarker v2.2. Before merging data, the same criteria were used to check the consistency of alleles, especially the alleles with small differences in fragment lengths.

The same individual for microsatellites was used for its mitochondrial COI amplification. As some individuals had not enough amount of DNA after microsatellites, the number of individuals in each population for COI data sets was unequal, between 10 and 31 individuals. The COI sequences were missed for two sampling sites in the dry season of Liuxihe reservoir. The mitochondrial gene COI was amplified using universal COI primers LCO1490 (5′-GGT CAA CAA ATC ATA AAG ATA TTG G-3′) and HCO2198 (5′-TAA ACT TCA GGG TGA CCA AAA AAT CA-3′; Folmer et al., 1994). The polymerase chain reactions were as follows in a total volume of 30 μl: 3 μl 10 × buffer (Mg^2+^ plus), dNTP Mixture 2.5 mM each, 0.5 μM of each primer, 0.5 U Taq, and 3 μl DNA template. The PCR conditions consisted of a 1 min initial cycle at 94 ^⁰^C, followed by 35 cycles of 40 s at 94^º^C, 40 s at 51^⁰^C, 60 s at 72^⁰^C, then a final extension of 3 min at 72^⁰^C, end of reaction at 4^⁰^C. The amplifications were verified and chosen for sequencing using a 1% agarose gel for electrophoresis. The PCR products were then sent to Huayu gene (Guangzhou, China) for sequencing on ABI3730 sequencer. All obtained sequences were checked for the absence of stop codons and ambiguous positions, and the validity of obtained sequences was verified by BLAST comparison in NCBI. The homologous alignment of sequences was performed using Aliview (Larsson, 2014). In total, all the obtained COI haplotypes were deposited in GenBank with numbers from ON734022 to ON734041.

### Genetic diversity and genetic structure

For microsatellites, genetic diversity was estimated in each population by GenALEx v6 (Peakall and Smouse, 2006), including the number of different alleles (Na), the number of effective alleles (Ne), expected (He), and observed (Ho) heterozygosity, the inbreeding coefficient (*F*_is_). *F*_is_ ranges from −1 to 1, where negative values significantly different from zero indicate an excess of heterozygotes and positive values indicate a deficiency of heterozygotes. The deviation from Hardy–Weinberg equilibrium was examined in Arlequin v3.5 (Excoffier and Lischer, 2010). As our populations all significantly deviated from the Hardy–Weinberg equilibrium that is required for STRUCTURE (Pritchard et al., 2000), the discriminant analysis of principal components (DAPC) was performed to investigate population genetic structure (Jombart et al., 2010). In DAPC, the genotype matrix was first transformed using principal component analysis (PCA), and then a linear discriminant analysis was performed on the retained principal components. DAPC analysis was implemented in R with the package adegenet (Jombart, 2008; Jombart et al., 2010).

For COI, the sequence characteristics and genetic diversity were examined with DnaSP v5.10 (Rozas et al., 2003), including the number of variable sites, haplotype diversity, and nucleotide diversity. To visually the relationships among the mitochondrial haplotypes, PopArt was used to construct the Minimum spanning network (Leigh and Bryant, 2015).

### Genetic differentiation and clustering analysis

The genetic differentiation (genetic distance) for COI was estimated in MEGA v6 (Kumar et al., 2008) with the Kimura 2-parameter model, and the bootstrap method was repeated 1,000 times. The pairwise-*F*_st_ for microsatellites was calculated to characterize genetic differentiation between populations. AMOVA in Arlequin v3.5 (Excoffier and Lischer, 2010) was used to partition the genetic variance into within seasons, among populations within seasons, and within populations. Due to missing COI sequences for two sampling sites in Liuxihe reservoir, AMOVA was only performed for the other three reservoirs. Temporal populations of 6 years were yearly grouped for AMOVA analysis to evaluate the interannual difference in genetic variation. The relationship between geographical distance and pairwise genetic distance (*F*_st_) within reservoirs was detected by a linear regression model with package ggplot2 (Wickham et al., 2016) in R v3.5.0 (R Core Team, 2018).

### Gene flow

Recent immigration rate over the last 3–5 generations and directional gene flow was estimated based on the principle of linkage disequilibrium with BayesAss v3.0 (Wilson and Rannala, 2003; Rannala, 2007), which employs Markov Chain Monte Carlo (MCMC) analysis. As BayesAss does not assume Hardy–Weinberg equilibrium, it was applied to identify the magnitude and direction of gene flow across spatial and temporal gradients/scales. The BayesAss MCMC was run for 10,000,000 iterations after an initial burn-in period of 10,000,000 iterations and sampled every 1,000 iterations.

## Results

### Spatial and temporal genetic variation based on microsatellites

One hundred and six (106) alleles were detected from 1,018 individuals of 32 populations. The average number of alleles (Na) of all populations was 3.96 (Variance = 0.79). All populations had low genetic diversity (Ho, Mean = 0.357, Variance = 0.086; He, Mean = 0.380, Variance = 0.074). There was no significant seasonal variation in genetic diversity within individual reservoirs. AMOVA showed that genetic variation was very low (< 7%) among populations within seasons and among seasons for each reservoir, and most of the genetic variance for the four reservoirs was contributed by within-population variation (87.9%–92.4%, Table 1).

**Table 1:** AMOVA of mtDNA and microsatellite datasets for testing the source of genetic variation.

	Chaishitan reservoir			Qiandaohu reservoir			Xujiahe reservoir		
	df	% variation	*p*-value	df	% variation	*p*-value	df	% variation	*p*-value
SSR									
Among seasons	1	3.75	*p* < 0.05	1	−0.1	*p* > 0.05	1	4.06	*p* < 0.05
Among populations within seasons	6	4.72	*p* < 0.05	6	7.66	*p* < 0.05	6	3.89	*p* < 0.05
within populations	502	91.53	*p* < 0.05	492	92.44	*p* < 0.05	504	92.05	*p* < 0.05
COI									
Among seasons	1	1.97	*p* < 0.05	1	10.64	*p* > 0.05	1	16.36	*p* < 0.05
Among populations within seasons	6	−2.08	*p* > 0.05	6	3.72	*p* > 0.05	6	2.8	*p* > 0.05
within populations	130	100.1	*p* > 0.05	167	85.64	*p* < 0.05	177	85.64	*p* < 0.05

Within each reservoir, the populations were sampled at four sites, and each site was sampled in two seasons.

**Figure 2:**
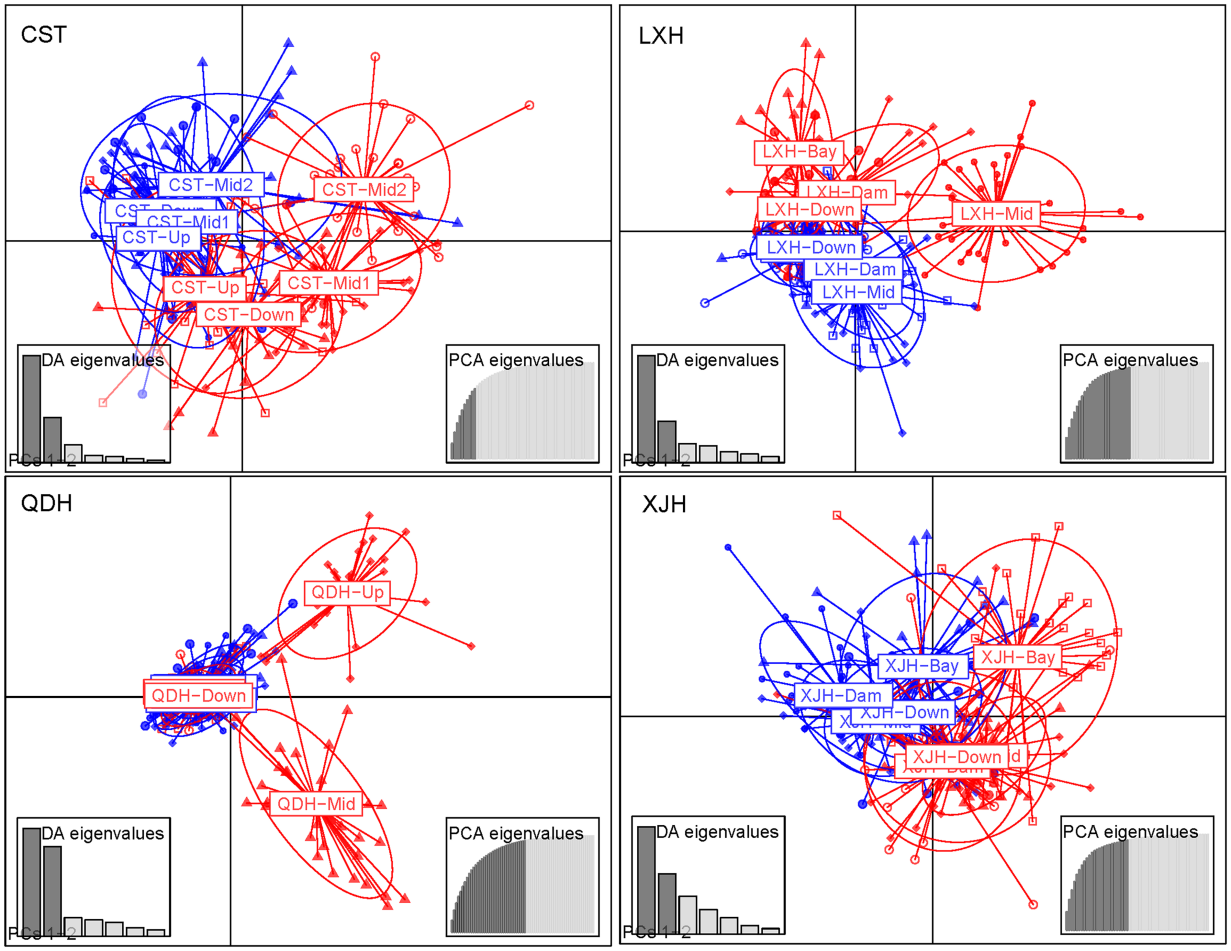
Discriminant Analysis of Principal Components (DAPC) analysis used to identify population genetic structure. The blue and red colors represent wet season and dry season, respectively.

**Figure 3:**
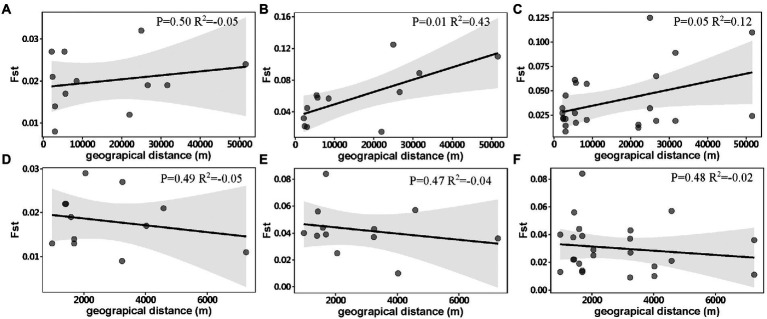
Relationship between genetic distance (*F*_st_) and geographical distance in Chaishitan and Qiandaohu reservoirs, **(A)** wet season, **(B)** dry season, **(C)** both seasons and Liuxihe and Xujiahe reservoirs, **(D)** wet season, **(E)** dry season, **(F)** both seasons. Each point indicates a site-pair within a single reservoir, any site-pair between two reservoirs was excluded. Gray area shows the 95% confidence interval level.

DAPC analysis revealed genetic differentiation among sampling sites within Chaishitan, Liuxihe, and Qiandaohu reservoirs (Supplementary Figures 1A,B,D,F). Such genetic differentiation varied temporally (Figure 2). In Liuxihe reservoir, a high genetic differentiation (*F*_st_ > 0.05) occurred in the dry season (i.e., between midstream (Mid) and bay (Bay)), between midstream (Mid) and downstream (Down), and low genetic differentiation (*F*_st_ < 0.05) did in the wet season (Supplementary Table 2). In Qiandaohu reservoir, except for one pair of sites, higher genetic differentiation (0.05 < *F*_st_ < 0.15) among sampling sites occurred in the dry season (Supplementary Table 2). No significant relationship was detected between genetic distance and spatial distance within single reservoirs. As Chaishitan and Qiandaohu reservoirs had the typical longitudinal gradient, a significant correlation was detected between *F*_st_ and spatial distance in the dry season by combining site pairs within each of the two reservoirs (*p* < 0.05, *R*^2^ = 0.43, Figure 3B) but not in the wet season (Figure 3A). However, by combining site pairs within Liuxihe reservoir and within Xujiahe reservoir, no significant relationship was detected between *F*_st_ and spatial distance in both seasons (Figures 3D–F).

### Spatial and temporal genetic variation based on COIs

We obtained 623 COI sequences with a length of 675 bp, including 138 from Chaishitan reservoir, 124 from Liuxihe reservoir, 171 from Qiandaohu reservoir, and 165 from Xujiahe reservoir. The sequences contained 37 variable sites and 20 haplotypes, indicating low clone diversity. The nucleotide diversity (Pi, Mean = 0.0066, Variance = 0.0048) and haplotype diversity (Hd, Mean = 0.41, Variance = 0.24) confirmed this. For Chaishitan, Liuxihe, and Xujiahe reservoirs, their genetic diversities all showed temporal and spatial differences (Supplementary Table 1).

**Figure 4:**
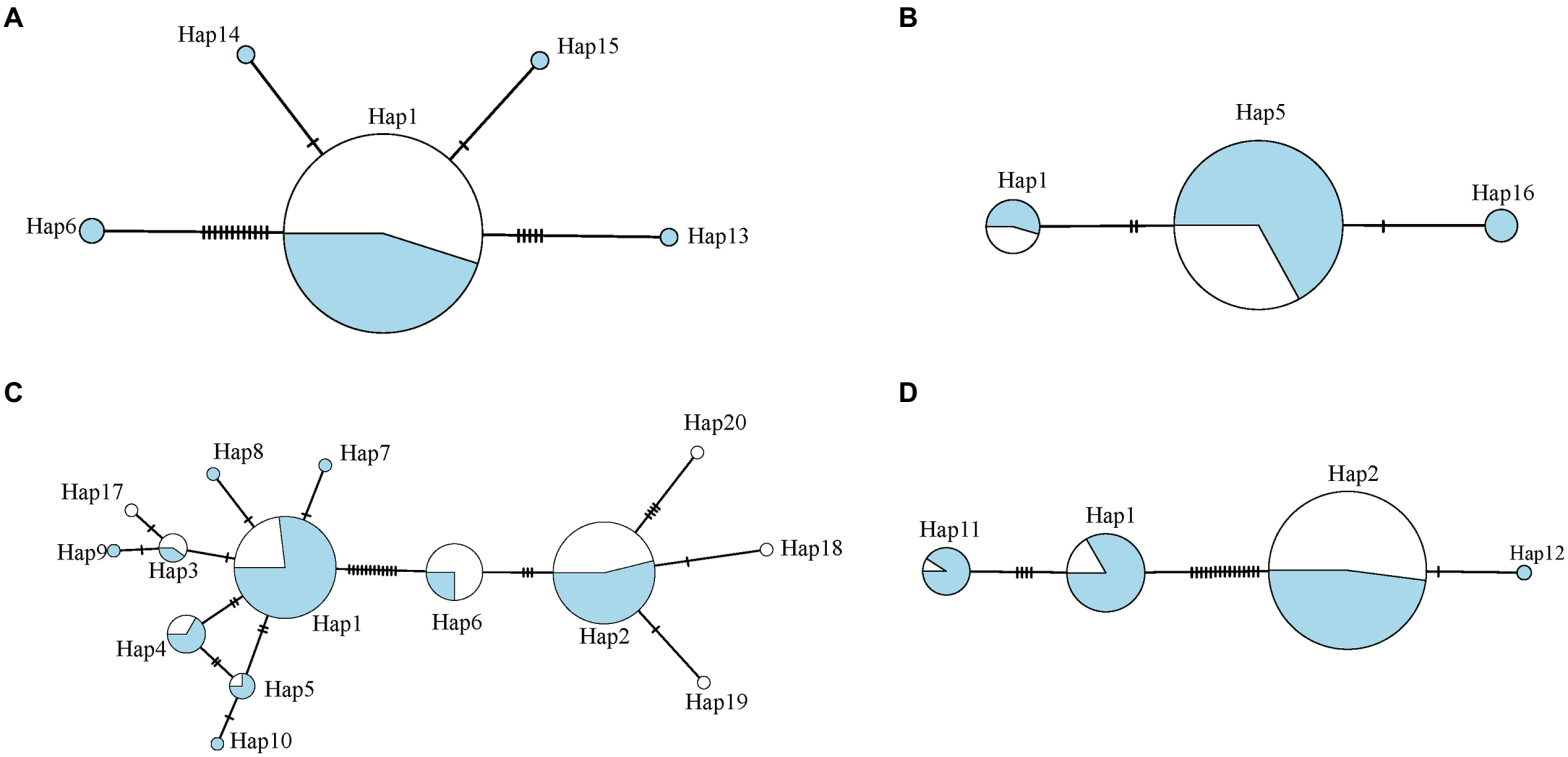
Haplotype networks for four reservoirs: **(A)** Chaishitan reservoir, **(B)** Liuxihe reservoir, **(C)** Qiandaohu reservoir, and **(D)** Xujiahe reservoir. Circle Size represents number of specimens with that haplotype, and blue indicates samples collected in the wet season and white indicates those in the dry season.

The haplotype networks revealed the temporal variation of clone composition (Figure 4). In Qiandaohu reservoir, rare haplotypes occurred only in one season (i.e., Hap 7, Hap 8, Hap 17, etc.), and major haplotypes seasonally changed in abundance, showing that Hap 2 dominated in the dry season, and Hap 1 and Hap 2 dominated in the wet season. In Chaishitan reservoir, five haplotypes were detected in the wet season, but one haplotype in the dry season, which was the main haplotype in the reservoir (Figure 4). In Liuxihe and Xujiahe reservoirs, Hap 16 and Hap 12 only appeared in the wet season.

AMOVA analysis showed that total population genetic variation in each reservoir was mainly from within populations (85.6%–100%, Table 1). Less genetic variation (1.97%, *p* < 0.05) between seasons occurred in Chaishitan reservoirs. The genetic variation between seasons was 10.64% (*p* > 0.05) and 16.36% (*p* < 0.05) in Qiandaohu and Xujiahe reservoirs, respectively. In the two reservoirs, the genetic distance showed weak population genetic differentiation (sequence differences >0.01) between the two sampling seasons.

### Temporal variation of genetic diversity in Liuxihe reservoir

One hundred and two (102) alleles were detected from 959 individuals of 32 temporal populations, and the average number of alleles (Na) for all populations was 3.52 (Variance = 0.10). The genetic diversity varied, and with a random seasonal pattern (Supplementary Figure 2). In 2014, the highest diversity appeared in the early growing season (March). In 2012, 2013, and 2017, genetic diversity peaked in the mid-late or mid of the growing season (June, May, or April). The expected heterozygosity (He) showed no significant interannual difference, while the average number of alleles (Na) had a significant interannual differences between 2012 and 2015 or between 2015 and 2017 (Supplementary Figure 3).

**Figure 5:**
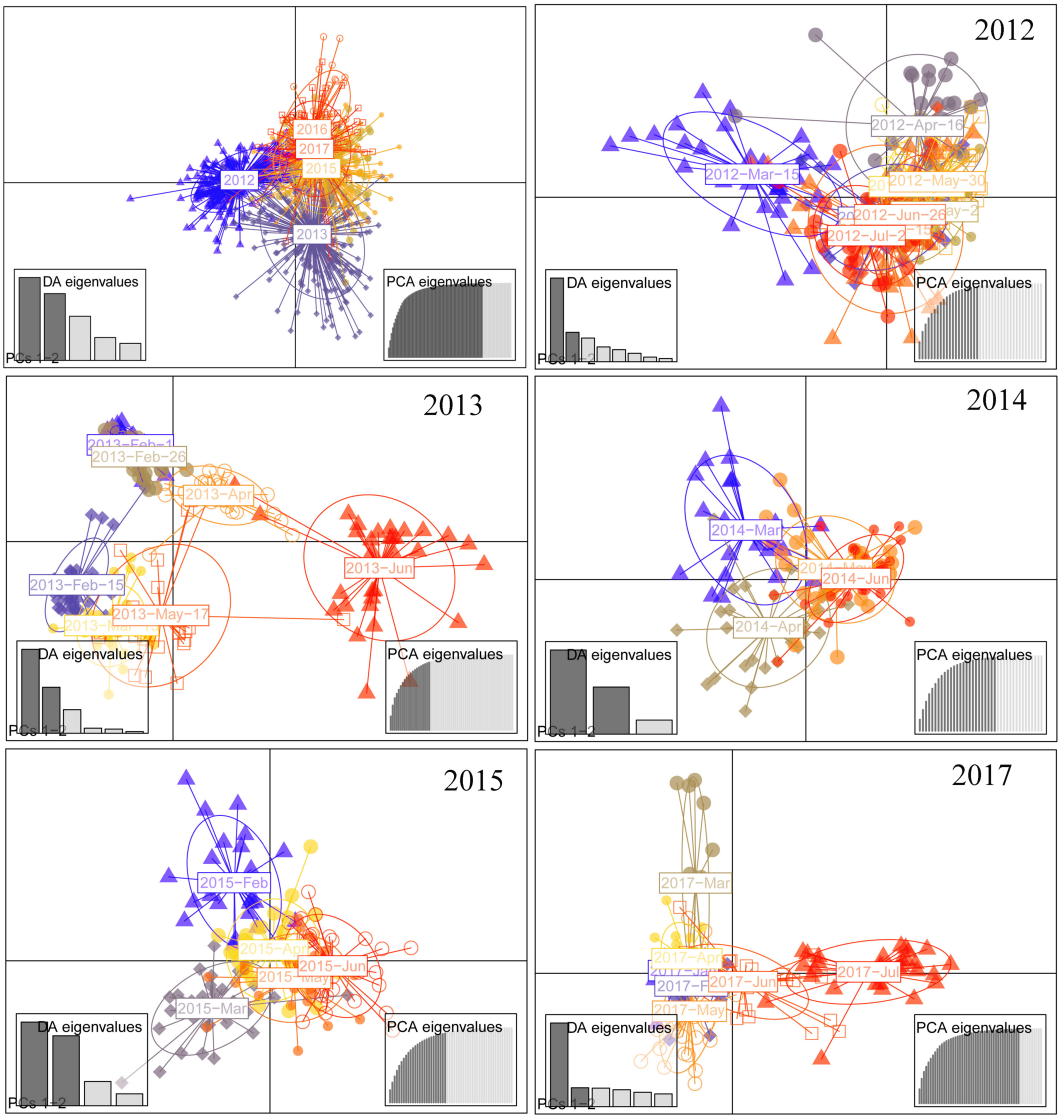
Scatterplot of DAPC analysis, used to identify genetic clusters for each year. Eigenvalue was shown as an inset for graph, with dark gray bars representing those used in the scatterplot.

DAPC revealed both annual and seasonal differences in genetic composition (Figure 5). Populations of 2012–2013 were separated from those of 2014–2017. Pairwise *F*_st_ between 6 years also showed low genetic difference (*F*_st_ < 0.05). AMOVA indicated that genetic variation was mainly from within populations (85.99%, *p* < 0.05), and less (3.9%) but significantly from interannual variation (*p* < 0.05; Table 2).

**Table 2:** AMOVA for temporal populations in Liuxihe reservoir.

Source of variation	d.f.	Sum of squares	Variance components	Percentage of variation	*p*-value
Among years	5	198.60	0.079	3.9	*p* < 0.05
Among populations within years	28	390.78	0.205	10.1	*p* < 0.05
Within populations	1,992	3,478.24	1.746	85.99	*p* < 0.05
Total	2,025	4,067.6	2.03		

Temporal genetic variation was higher in 2013 and 2017 (Figure 5). Genetic differentiation (*F*_st_) between temporal populations had a mean of 0.088 with a variance of 0.031 in 2013. High genetic differentiation occurred between July and other months in 2017 (Supplementary Table 3). Rather low genetic differentiation (*F*_st_ < 0.05) occurred in 2014 and also in 2015 except for two pairs of temporal populations in the year (Supplementary Table 3).

### Gene flow within reservoirs

**Figure 6:**
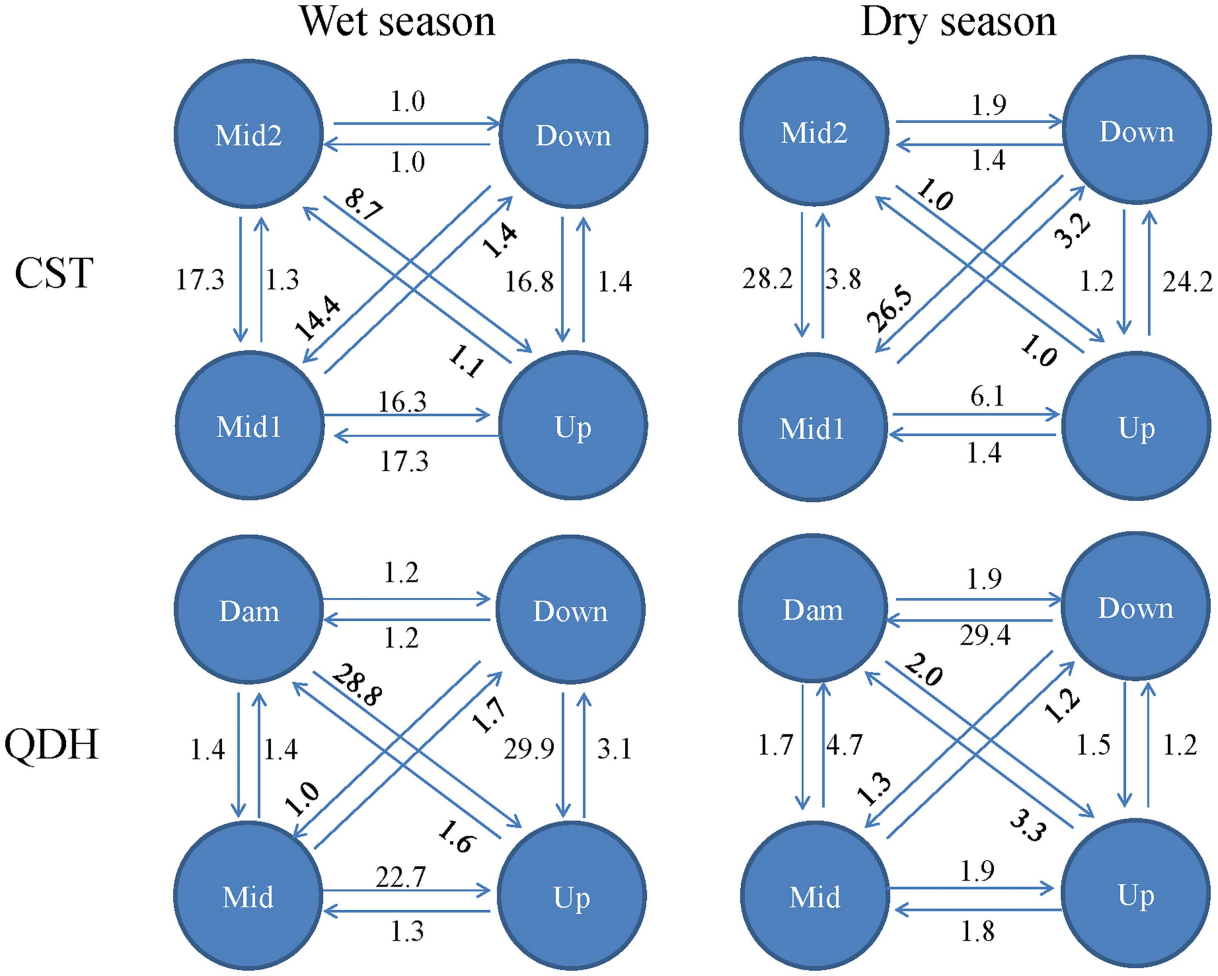
Gene flow between populations within two reservoirs characterized by a typical longitudinal environmental gradient. CST: Chaishitan reservoir; QDH: Qiandaohu reservoir. Up: upstream; Mid: midstream; Down: downstream, Dam: near the dam. Upstream of both reservoirs belonged to riverine zone, midstream of both reservoirs and downstream of Qiandaohu reservoir belonged to transitional zone, downstream of Chaishitan reservoir and open water near the dam of Qiandaohu reservoir belonged to lacustrine zone.

In the wet season, the direction of gene flow in Chaishitan reservoir was from the lacustrine zone to the transitional zone and to the riverine zone (from Down to Up, from Mid2 to Mid1 or Up). And there was symmetrical and high gene flow between the transitional zone to the riverine zone (Mid1 and Up). In the dry season, gene flow was from the riverine zone to the lacustrine zone (from Up to Down), as well as from the transitional zone or the lacustrine zone to riverine zone (from Mid2 to Mid1, from Down to Mid1). In the wet season in Qiandaohu reservoir, high gene flow mainly occurred from the lacustrine zone or transitional zone to the riverine zone (Figure 6). Compared to the wet season, the spatial gene flow was weakened in the dry season, and there was strong gene flow from the transition zone to lacustrine zone.

For annual populations across 6 years in the Liuxihe reservoir, both forward (from 2013 to 2014) and backward gene flows (from 2013 to 2012, 2015 to 2014, 2016 to 2015, and 2017 to 2016) were detected (Supplementary Figure 4). For temporal populations of each year, the detected backward gene flows mainly occurred in the mid-late growing season or/and the early growing season (i.e., from May 2012 to April 2012, April 2015 to May 2015, and Jan 2017 to Feb 2017; Supplementary Figure 4).

## Discussion

The present study investigated temporal and spatial fine-scale variation in genetic diversity and structure of the *D. galeata* populations. Spatial fine-scale variation occurred and changed between two sampling seasons, especially in the two reservoirs (Chaishitan and Qiandaohu reservoirs) with longitudinal gradients. Genetic differentiation increased with spatial distance in the dry season, indicating increased environmental selection. Seasonal variation of genetic diversity at a pelagic site of Liuxihe reservoir appears to peak in the mid or mid-late growing season and did not follow an erosion pattern.

### Spatial variation of genetic structure

Clear spatial genetic variation was observed in the Chaishitan and Qiandaohu reservoirs with a typical longitudinal environmental gradient. Higher genetic differentiation occurred even between the transitional zone and riverine zone in the Qiandaohu reservoir (*F*_st_ > 0.1, Supplementary Table 2). A significant correlation between *F*_st_ and geographic distance (Figure 3) demonstrates that spatial differentiation was induced by environmental selection rather than by restricted gene flow. Environmental selection was considered the driver of spatial variation in the genetic structure, directly, and indirectly altering population genetic composition (Weider et al., 2005; Hembre and Megard, 2006; Brzeziński et al., 2010; Orsini et al., 2013; Maruki et al., 2019). In a reservoir characterized by a strong longitudinal environmental gradient, significant spatial differences in plankton structure and fish density were frequently detected (Vašek et al., 2004; Yang et al., 2018; Rizo et al., 2020). As an epilimnetic species, less vertical migration lets *D. galeata* be exposed to stronger predation or/and low food quality, which facilitates its genetic differentiation (Weider et al., 2005; Hembre and Megard, 2006; Brzeziński et al., 2010; Maruki et al., 2019). Large reservoirs usually have high longitudinal heterogeneity, which also explained the greater spatial variation of genetic structure in the Qiandaohu than in the Chaishitan reservoir (Figure 2). Fine-scale genetic differentiation of *D. galeata* was well investigated in a study in Rimov reservoir of the Czech Republic, and significant intraspecific genetic differentiation was detected between the upstream and downstream of the reservoir (Petrusek et al., 2013). Even the hypolimnion population was genetically differentiated from the epilimnetic population (Seda et al., 2007). The observed vertical differentiation primarily resulted from fish predation pressure.

In Liuxihe and Xujiahe reservoirs, the spatial genetic differentiation was weaker. Although the two reservoirs have morphologically longitudinal zonation, their riverine zones are too short and shallow for *D. galeata* to have a stable population across seasons. The riverine zone was not sampled for the two reservoirs. Indeed, a similar genetic structure occurred across sites in Xujiahe reservoir and there was no significant correlation between *F*_st_ and geographic distance. Some earlier studies also did not detect strong population differentiation within lakes (Wolf, 1985; Carvalho and Crisp, 1987). A random spatial distribution of genotypes was detected for the haplophilic zooplankter *Artemia urmiana* from 15 different spatial sites in Lake Urmia, due to a lack of salinity differentiation in this lake (Eimanifar and Wink, 2013). Even without strong environmental difference between populations within a landscape, some genetic differentiation can arise due to purely stochastic processes, given the spatial separation of the two populations (Gillespie, 2004; Petrusek et al., 2013). In Liuxihe reservoir located near to the Tropic of Cancer, *D. galeata* had a smaller population size and poorer haplotypes than in the other reservoirs. Small effective population size increases genetic drift and population differentiation (Freeman and Herron, 2004; Vanoverbeke et al., 2007). Stochastic effects associated with hatching from resting egg banks combined with genetic drift can lead to significantly differentiated active populations, even if the genetic composition of their resting egg banks was identical (Schwentner and Richter, 2015).

The population differentiation between the two sampling sites was not permanent within any reservoir investigated here. Any spatial pattern for environmental selection can be disrupted by seasonal changes in food resources, predators, or abiotic factors (Frisch and Weider, 2010; Yin et al., 2012; Petrusek et al., 2013). Large reservoirs are commonly built for flooding control and irrigation, their water level fluctuates seasonally and largely depends on water use. Water level fluctuation was found to be an important factor influencing spatial genetic variation within a single water body (Sturmbauer et al., 2001; Nevado et al., 2013). Qiandaohu reservoir is located in the lower reaches of the Yangtze River, and its precipitation is concentrated in wet seasons, especially from March to June (Liu et al., 2020). During this period, the water level fluctuates greatly, which temporarily disrupts the established environmental gradients, increasing gene flow along the direction of water flow and weakening spatial genetic variation (Figure 6; Supplementary Figure 1). The water level fluctuation and environmental conditions tend to be stable in dry seasons, during which a longitudinal environmental gradient is established and significantly decreased gene flow and increases genetic differentiation between populations. As a result, spatial genetic structure can be detected in dry season. Interestingly, spatial genetic structure was detected in both sampling seasons in Chaishitan reservoir. The reservoir is located in Yunnan-Guizhou Plateau, and had low precipitation and water level fluctuation. A stronger spatial genetic variation was observed in the dry season. Such seasonal change in spatial variation within a water body was also observed in Rimov reservoir (Petrusek et al., 2013), in which a significant spatial differentiation of *D. galeata* occurred at more than half of the sampling dates. In Lake Texoma, significant spatial heterogeneity of genotype frequencies was observed in *D*. *lumholtzi*, but restricted to the summer (Frisch and Weider, 2010). The composition of *D. galeata* genotypes also changed seasonally. Although some clones (haplotypes) occurred in the two sampling seasons, their relative frequencies often differed (Figure 4). The frequency of each clone fluctuated on a time scale, which presumably reflected environmental change (Carvalho and Crisp, 1987). Seasonal change in environmental conditions affects competition between clones, and leads to a shift in genotype composition (Stibor and Lampert, 2000; Yin et al., 2012).

### Temporal variation of genetic diversity and structure in Liuxihe reservoir

In temperate region, cyclic parthenogenetic zooplankton are characterized by high genetic diversity in the initial growing season, which is rapidly established from a dormant egg bank (De Meester et al., 2006; Rother et al., 2010). During the growing seasons, however, selection pressure and genetic drift are expected to erode genetic diversity within a population, *viz.*, a decline in genetic diversity over time (Ortells et al., 2006). Towards the next initial growing season, genetic diversity is re-established from the dormant egg bank (De Meester et al., 2006). The local genetic diversity of *D. galeata* in Liuxihe reservoir did not show this theoretical erosion pattern, but seems to be a random pattern. And the genetic diversity of Liuxihe reservoir appears to peak slightly in mid or mid-late growing season, rather than in early growing season as temperate region. The main growth phase of *D. galeata* in tropical China was from December to July. In early spring that starts from the December, low water temperature (about 14^⁰^C) is suitable for the hatching of dormant eggs, and individuals quickly re-established from the egg bank (Vandekerkhove et al., 2005). During the mixing period, dormant eggs have chances to be suspended in shallow zones. The observed backward gene flow supports genotypes or clones recruited from the resting egg bank in Jan–March (Supplementary Figure 4). Genetic diversity of *D. galeata* in Liuxihe reservoir was not only higher but also stable from May to June. The deep zone in a reservoir usually serves as a sink that accumulates more genotypes (Yin et al., 2012). During seasonally flooding, the pelagic zone collected genotypes or clones imported from shallow waters where resting eggs may hatch. Newly established genotypes would come from upstream zones, but there was not indication that hydrological conditions could explain a higher import (Hulsmann et al., 2012). Although re-hatched from resting eggs may contribute to genetic diversity, we did not detect the backward gene flow during the flood season, at least in 2017. From June to July, the genetic diversity of *D. galeata* decreased with declining population abundance in Liuxihe reservoir, indicating that increasing fish predation functions as the selection pressure eroding clonal diversity (Vanoverbeke and De Meester, 2010).

Genetic variation of *Daphnia* populations is largely controlled by selection acting on individuals recruited periodically from dormant populations (Hembre and Megard, 2006). We detected significant seasonal variation in genetic structure in 2013 and 2017. Especially in 2013, there was large genetic differentiation (0.05 < *F*_st_ < 0.15) between most temporal populations. This seasonal differentiation may suggest recruitment and selection acting together, and which usually occurred in early or end growing season. The contribution of resting eggs to the population may profoundly altered the genetic composition of the population compared to the previous season (Hulsmann et al., 2012). In end growing season, the increasing fish population results in stronger predation pressure, which lead to genetic differentiation of *Daphnia* populations (Hembre and Megard, 2006). Similarly, due to intense clonal selection, the parthenogenetic population of *Myzus persicae* was characterized by strong and rapid change in the relative frequencies of common clones during the course of a year (Vorburger, 2006). And significant temporal heterogeneity of *D. lumholtzi* existed in genotype frequencies with a major shift only between summer and autumn (Frisch and Weider, 2010). Population genetic differentiation was detected between July and the other periods in 2017. Interestingly, there was no significant difference in the genetic diversity and genetic structure between years in the Liuxihe reservoir. In this case, the resting egg bank may buffer genetic diversity (Marshall, 2016; Orsini et al., 2016).

### The implication for population genetics and phylogeographic studies

In our species, temporal and fine-scale genetic variations can occur within a single water body, especially in large reservoirs. Consequently, exploring population genetic structure at a regional scale requires the organisms and their populations are reasonably collected to avoid high temporal and fine-scale spatial genetic variation. In practical sampling surveys, however, the populations usually comprise the samples collected from several seasons (i.e., White et al., 2010; Xu et al., 2011; Liu et al., 2018), and such a sampling strategy might lead to an underestimation of genetic variation between spatial samples (Balloux and Lugon-Moulin, 2002). Mitochondrial COI records are more useful in gathering historical and geographic information (i.e., Hebert et al., 2003; Bekker et al., 2018), and are widely used for phylogeographic studies (i.e., De Gelas and De Meester, 2005; Bekker et al., 2018). Phylogeographic studies without sufficient sampling across a specific geographic area are prone to incomplete and spurious patterns (Avendaño et al., 2017). Such phylogeographic studies typically encompass a large scale, and thus overlook temporal variation. The sampling strategy reflects the common assumption that the observed genetic structure and diversity remain temporally stable (Garant et al., 2000; Arnaud and Laval, 2004). Genetic drift in small populations can result in significant genetic differentiation between seasonal populations (Gómez et al., 1995; Freeman and Herron, 2004; Frisch and Weider, 2010). Temporal variation of genetic diversity and clonal composition occurs commonly in our studies, and high genetic diversity was detected in the mid or mid-late of the growing season. The hierarchical AMOVA demonstrated that temporal differentiation was higher than spatial differentiation (i.e., Xujiahe reservoir). The seasonal variation between populations can possibly mask the true geographical patterns (Xiang et al., 2015). Therefore, in addition to covering large geographical ranges, phylogeographic studies also need to consider potential temporal variation, especially for those waterbodies hosting deep and rare lineages. For zooplankton of cyclical parthenogens, samples are suggested to be collected in early or the mid of the growing season in exploring population genetics and genetic diversity under limited resources. Local environmental variables that were ignored in the present survey are strongly suggested to be recorded and measured for examining the expected environmental selection.

## Data availability statement

The datasets presented in this study can be found in online repositories. The names of the repository/repositories and accession number(s) can be found at: https://www.ncbi.nlm.nih.gov/genbank/, ON734022–ON734041 https://figshare.com/, 10.6084/m9.figshare.21321264.

## Author contributions

LeX and B-PH conceived and designed the experiments. QH, LeX, and LiX performed the experiments and analyzed and discussed the results. QH and B-PH wrote the manuscript. PL, ER, and B-PH revised the manuscript. All authors contributed to the article and approved the submitted version.

## Funding

The study was supported by a grant from National Natural Science Foundation of China (32171538 and 31400344) and the open grant (2022-2) from Engineering Research Center of Tropical and Subtropical Aquatic Ecological Engineering, Chinese Ministry of Education.

## Conflict of interest

The authors declare that the research was conducted in the absence of any commercial or financial relationships that could be construed as a potential conflict of interest.

## Publisher’s note

All claims expressed in this article are solely those of the authors and do not necessarily represent those of their affiliated organizations, or those of the publisher, the editors and the reviewers. Any product that may be evaluated in this article, or claim that may be made by its manufacturer, is not guaranteed or endorsed by the publisher.
