# Supplementary material for: Spatial and temporal variation of genetic diversity and genetic differentiation in Daphnia galeata populations in four large reservoirs in southern China
Source: Front Microbiol. 2022 Nov 11;13:1041011. doi: 10.3389/fmicb.2022.1041011 (PMC9691881; doi:10.3389/fmicb.2022.1041011)
Supplement: Supplementary file 1 [file Data_Sheet_1.PDF]

Supplementary Material

Supplementary Table 1. Genetic diversity of *Daphnia galeata* measured with microsatellites and mitochondrial COI

Reservoir	Sampling period	Site	Microsatellites					COI			
			N	Na	Ho	He	F_{is}	N_{seq}	N_{hap}	Hd	Pi (%)
CST	Wet season	Mid2-1	32	4.889	0.368	0.429	0.271	19	3	0.205	0.265
		Down-1	32	3.444	0.444	0.416	0.033	17	3	0.228	0.035
		Mid1-1	32	3.778	0.431	0.419	0.082	17	2	0.118	0.209
		Up-1	32	4.111	0.503	0.396	-0.096	12	1	0	0
	Dry Season	Mid2-2	32	3.667	0.486	0.401	-0.087	18	1	0	0
		Down-2	31	3.778	0.355	0.346	-0.025	24	1	0	0
		Mid1-2	32	3.444	0.441	0.380	-0.044	17	1	0	0
		Up-2	32	4.222	0.535	0.494	-0.098	14	1	0	0
LXH	Wet season	Bay-2	32	3.667	0.316	0.351	0.239	15	2	0.248	0.030
		Dam-2	32	3.444	0.285	0.348	0.209	25	3	0.227	0.057
		Down-2	32	3.111	0.319	0.316	0.071	17	2	0.118	0.035
		Mid-2	32	3.333	0.323	0.352	0.311	26	3	0.280	0.074
	Dry season	Bay-1	32	2.111	0.212	0.208	0.106	17	2	0.118	0.035
		Dam-1	32	3.444	0.243	0.351	0.395	-	-	-	-
		Down-1	29	3.000	0.218	0.282	0.227	-	-	-	-
		Mid-1	36	4.111	0.352	0.363	0.032	24	2	0.290	0.086
QDH	Wet season	Dam-1	32	5.222	0.410	0.452	0.175	23	4	0.628	1.104
		Down-1	32	5.333	0.448	0.459	0.070	28	6	0.672	1.021
		Mid-1	32	5.444	0.424	0.479	0.094	28	5	0.698	1.215
		Up-1	32	4.222	0.427	0.467	0.084	21	5	0.655	1.193
	Dry season	Dam-2	32	5.111	0.330	0.413	0.228	23	6	0.601	0.819
		Down-2	32	5.000	0.378	0.479	0.210	18	4	0.595	0.763

		Mid-2	32	5.000	0.431	0.516	0.197	20	7	0.816	1.268
		Up-2	26	4.111	0.385	0.451	0.157	10	5	0.756	1.215
XJH	Wet season	Bay-1	32	3.444	0.333	0.346	0.017	21	3	0.600	1.244
		Mid-1	32	3.444	0.333	0.315	0.024	22	4	0.636	1.240
		Down-1	32	3.889	0.330	0.331	0.109	21	3	0.602	1.263
		Dam-1	32	4.000	0.330	0.325	0.127	31	2	0.361	0.803
	Dry season	Bay-2	32	2.889	0.205	0.228	0.119	17	1	0	0
		Mid-2	32	3.889	0.236	0.376	0.331	19	2	0.281	0.624
		Down-2	32	3.667	0.288	0.332	0.248	16	2	0.125	0.315
		Dam-2	32	4.556	0.299	0.335	0.178	18	2	0.209	0.465

Abbreviation: N, number of individuals; Na, number of alleles; Ho, observed heterozygosity; He, expected heterozygosity; F_{is}, inbreeding coefficient; N_{seq}, number of sequenced individuals; N_{hap}, number of haplotypes; Hd, haplotype diversity; Pi, nucleotide diversity. Up: upstream; Mid: midstream; Down: downstream; Dam: near the dam, Bay: a semi close coastal water, "-": missing data.

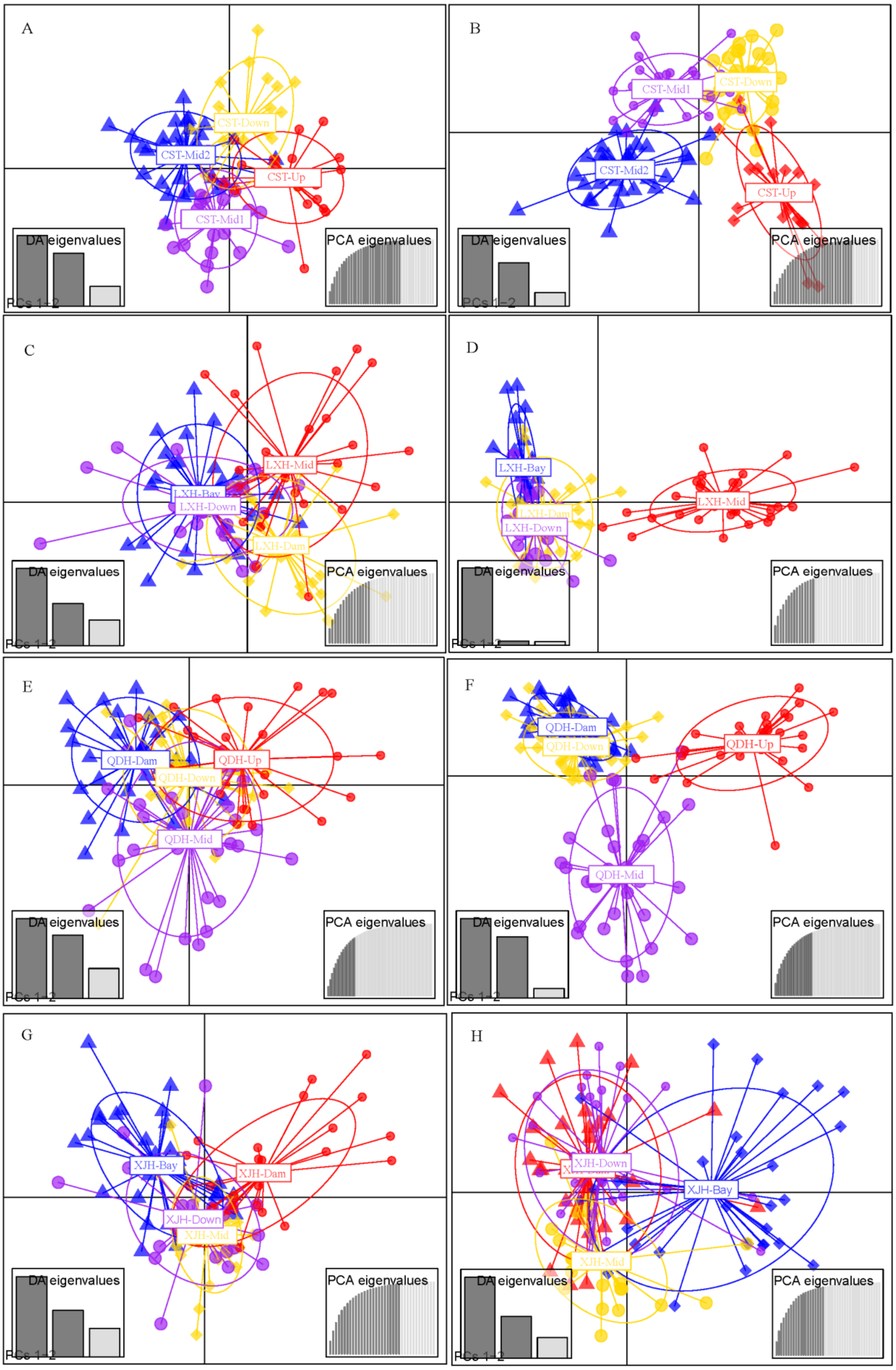
A
CST-Down
CST-Mid2
CST-Up
CST-Mid1
DA eigenvalues
PCA eigenvalues
PCs 1-2
B
CST-Mid1
CST-Down
CST-Mid2
CST-Up
DA eigenvalues
PCA eigenvalues
PCs 1-2
C
LXH-Mid
LXH-Bay
LXH-Down
LXH-Dam
DA eigenvalues
PCA eigenvalues
PCs 1-2
D
LXH-Bay
LXH-Dam
LXH-Down
LXH-Mid
DA eigenvalues
PCA eigenvalues
PCs 1-2
E
QDH-Dam
QDH-Up
QDH-Down
QDH-Mid
DA eigenvalues
PCA eigenvalues
PCs 1-2
F
QDH-Dam
QDH-Down
QDH-Up
QDH-Mid
DA eigenvalues
PCA eigenvalues
PCs 1-2
G
XJH-Bay
XJH-Dam
XJH-Down
XJH-Mid
DA eigenvalues
PCA eigenvalues
PCs 1-2
H
XJH-Down
XJH-Bay
XJH-Mid
DA eigenvalues
PCA eigenvalues
PCs 1-2

Supplementary Figure 1. Discriminant Analysis of Principal Components (DAPC) analysis of microsatellite data used to identify genetic clusters. The axes represent the first two linear discriminants. Each dot indicates an individual and cluster are shown by different colors and ellipses. The left bottom scuttle shows the DA eigenvalue of each linear discriminants, the right bottom scuttle displays the cumulative variance of the PCA axis. A and B: samples from Chaishitan reservoir in wet season and dry season, respectively; C and D: samples from Liuxihe reservoir in wet season and dry season, respectively; E and F: samples from Qiandaohu reservoir in wet season and dry season respectively; G and H: samples from Xujiahe reservoir in wet season and dry season, respectively.

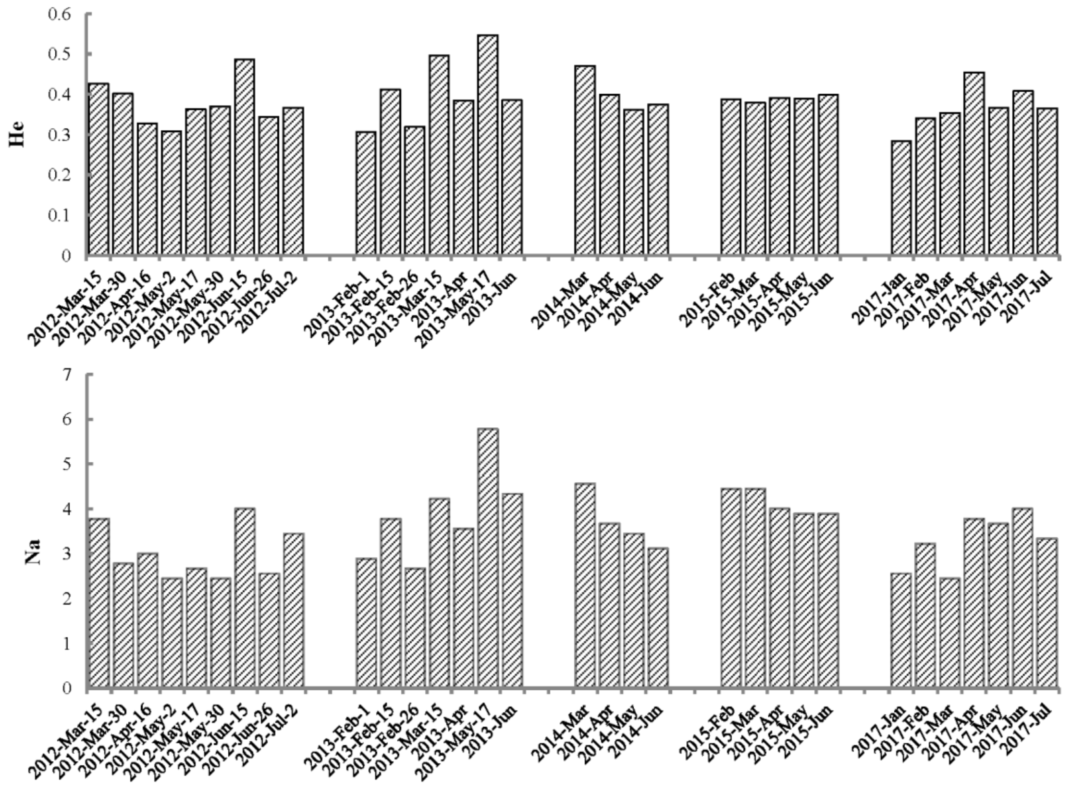

Supplementary Figure 2. Temporal dynamics of genetic diversity at a pelagic zone in Liuxihe reservoir: expected heterozygosity (He) and average number of alleles (Na).

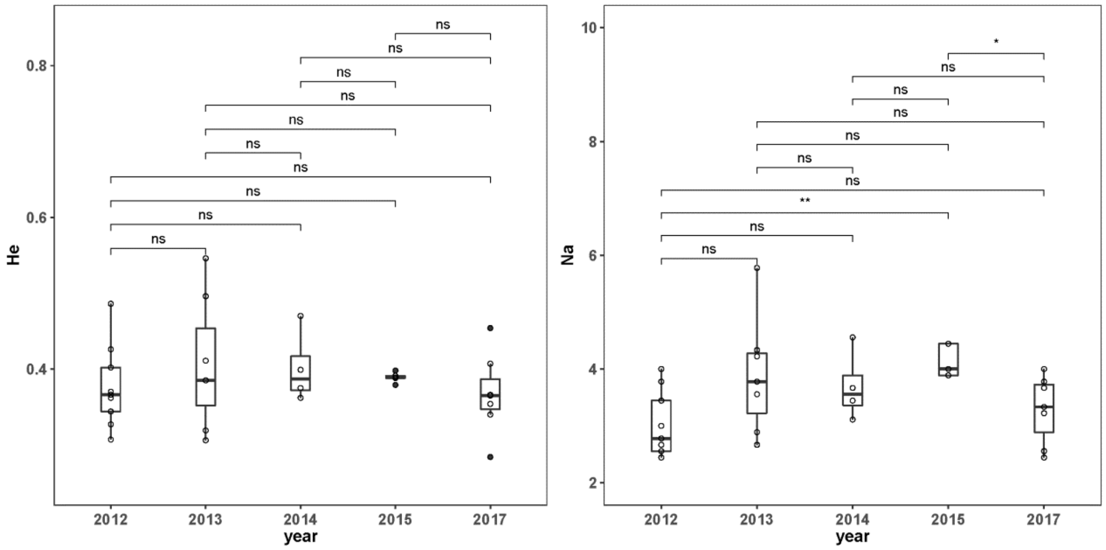

Supplementary Figure 3. The interannual variation of expected heterozygosity (He) and average number of alleles (Na) in Liuxihe reservoir. *: $P < 0.05$, **: $P < 0.01$, ns: $P > 0.05$.

Supplementary Table 2. Pairwise F_{st} within reservoirs in dry season (below diagonal) and in wet season (above diagonal). a: Chaishitan reservoir, b: Liuxihe reservoir, c: Qiandaohu reservoir, d: Xujiahe reservoir.

a

	Mid2	Down	Mid1	Up
Mid2		0.027	0.021	0.027
Down	0.027		0.008	0.017
Mid1	0.021	0.008		0.014
Up	0.027	0.017	0.014	

b

	Bay	Dam	Down	Mid
Bay		0.038	0.040	0.084
Dam	0.022		0.025	0.044
Down	0.013	0.029		0.056
Mid	0.014	0.019	0.022	

c

	Dam	Down	Mid	Up
Dam		0.015	0.065	0.110
Down	0.012		0.057	0.089
Mid	0.019	0.020		0.125
Up	0.024	0.019	0.032	

d

	Bay	Mid	Down	Dam
Bay		0.057	0.039	0.043
Mid	0.021		0.037	0.036
Down	0.013	0.009		0.010
Dam	0.027	0.011	0.017	

Supplementary Table 3. Pairwise F_{st} within years based on microsatellites.

2012

	Mar-15	Mar-30	Apr-16	May-2	May-17	May-30	Jun-15	Jun-26	Jul-2
Mar-15									
Mar-30	0.064								
Apr-16	0.078	0.032							
May-2	0.135	0.054	0.050						
May-17	0.099	0.035	0.027	0.034					
May-30	0.129	0.051	0.043	0.022	0.033				
Jun-15	0.063	0.016	0.040	0.053	0.034	0.050			
Jun-26	0.069	0.021	0.024	0.041	0.033	0.052	0.029		
Jul-2	0.050	0.028	0.033	0.055	0.041	0.067	0.033	0.013	

2014

	Mar	Apr	May	Jun
Mar				
Apr	0.017			
May	0.018	0.024		
Jun	0.019	0.025	0.011	

2015

	Feb	Mar	Apr	May	Jun
Feb					
Mar	0.041				
Apr	0.025	0.030			
May	0.037	0.036	0.012		
Jun	0.057	0.052	0.025	0.020	

2013

	Feb-1	Feb-15	Feb-26	Mar-15	Apr	May-17	Jun
Feb-1							
Feb-15	0.078						
Feb-26	0.031	0.081					
Mar-15	0.131	0.070	0.113				
Apr	0.078	0.129	0.069	0.075			
May-17	0.121	0.079	0.094	0.038	0.057		
Jun	0.139	0.133	0.105	0.102	0.062	0.062	

2017

	Jan	Feb	Mar	Apr	May	Jun	Jul
Jan							
Feb	0.041						
Mar	0.052	0.027					
Apr	0.054	0.021	0.032				
May	0.023	0.024	0.040	0.030			
Jun	0.053	0.031	0.042	0.026	0.021		
Jul	0.123	0.062	0.082	0.061	0.084	0.058	

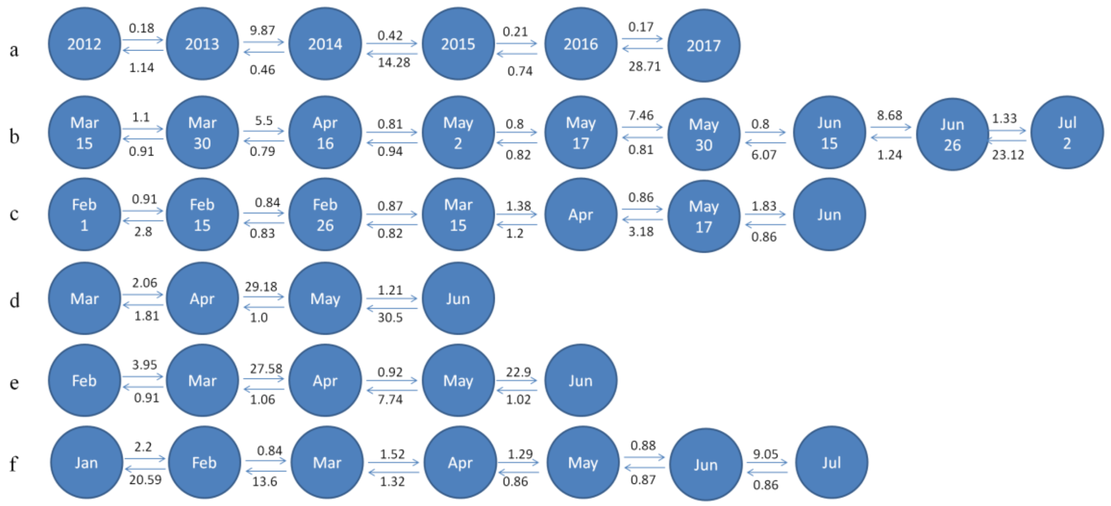

Supplementary Figure 4. Gene flow between populations across temporal in Liuxihe reservoir. The magnitude of gene flow was shown in percentage. a: between years; b: in 2012; c: in 2013; d: in 2014; e: in 2015; f: in 2017.
